# Urobilin Derived from Bilirubin Bioconversion Binds Albumin and May Interfere with Bilirubin Interacting with Albumin: Implications for Disease Pathology

**DOI:** 10.3390/biomedicines13020302

**Published:** 2025-01-26

**Authors:** Kevin I. Williams, Priyanka Suryadevara, Chang-Guo Zhan, Terry D. Hinds, Zachary A. Kipp

**Affiliations:** 1 Drug & Disease Discovery D3 Research Center, Department of Pharmacology and Nutritional Sciences, University of Kentucky College of Medicine, Lexington, KY 40508, USA; kevin.williams@centre.edu; 2 Department of Biochemistry and Molecular Biology, Centre College, Danville, KY 40422, USA; 3 Department of Pharmaceutical Sciences and Center for Pharmaceutical Research and Innovation, College of Pharmacy, University of Kentucky, Lexington, KY 40508, USA; suryadevarapriyanka@uky.edu (P.S.); chang-guo.zhan@uky.edu (C.-G.Z.); 4 Markey Cancer Center, University of Kentucky, Lexington, KY 40508, USA; 5 Barnstable Brown Diabetes Center, University of Kentucky, Lexington, KY 40508, USA

**Keywords:** autofluorescence, albumin, heme oxygenase, PPAR, biliverdin reductase, bilirubin reductase, urobilinogen, stercobilin, biliverdin

## Abstract

**Background/Objectives:** Bilirubin is a hydrophobic molecule that binds the carrier protein albumin for transport through systemic circulation. Bilirubin is cleared from the body through the liver and excreted into the intestines, where the microbiota modifies the chemical structure, forming urobilin, which can be reabsorbed into circulation by the hepatic portal vein. Urobilin has no known function. It is also unknown whether urobilin binds albumin for transport in circulation. We hypothesized that because of the likeness of their chemical structures, urobilin would also bind albumin like bilirubin does. **Methods:** First, we used in silico docking to predict if urobilin would bind to albumin and compared it to the bilirubin binding sites. To test this binding in vitro, we applied bilirubin’s fluorescent property, which occurs when it is bound to a protein, including albumin, and exposed to light. We also used this method to determine if urobilin could exhibit autofluorescence when protein bound. **Results:** We found that bilirubin was predicted to bind albumin at amino acids E208, K212, D237, and K240 through hydrogen bonds. However, urobilin was predicted to bind albumin using different hydrogen bonds at amino acids H67, K240, and E252. We found that urobilin has a fluorescent property that can be quantified when bound to albumin. We performed a concentration response for urobilin–albumin fluorescent binding and observed a direct relationship between the urobilin level and the fluorescence intensity. **Conclusions:** The in silico docking analysis and autofluorescence results demonstrate that urobilin binds to albumin and might compete with bilirubin. This is the first study to identify a urobilin-binding protein and the important aspects of its physiological function and transport in circulation.

## 1. Introduction

The breakdown of red blood cells commences the production of bilirubin from heme [1], which ultimately travels to the intestine and is converted to urobilin [2]. Bilirubin is protective against cardiometabolic diseases [3,4], including obesity [5], diabetes [6], hypertension [7,8], and metabolic dysfunction-associated steatotic liver disease (MASLD) [9]. Bilirubin functions as a potent antioxidant [10] and also as a hormone by activating the nuclear receptor peroxisome proliferator-activated receptor alpha (PPARα) [11,12,13,14,15]. Bilirubin is produced mostly in the spleen, which enters systemic circulation to reach its target organs. Due to its hydrophobic nature, bilirubin is tightly bound to albumin in plasma as a carrier protein [16]. Once in the liver, bilirubin is conjugated by UDP glucuronosyltransferase 1 (UGT1A1), which increases bilirubin’s solubility for excretion through the biliary system into the intestines [17]. In the gut, bacteria remove the conjugation and change the chemical structure to form urobilinogen, which is rapidly oxidized into urobilin [2]. Hall et al. discovered the enzyme responsible for the bioconversion of bilirubin to urobilinogen in the bacterial genome, bilirubin reductase (BilR), which is primarily in the *Firmicutes* species [18]. Urobilin can be reabsorbed through the hepatic portal system into the systemic circulation. Unlike bilirubin, urobilin has been positively associated with adiposity, insulin resistance, and cardiovascular disease [2,19,20,21]. The physiological function of urobilin and whether it utilizes albumin as a carrier protein for transport in circulation is unknown.

Bilirubin has a natural autofluorescent property when bound to a protein, which has been well documented for the bilirubin–albumin interaction [22,23,24,25]. A study published in 1973 by Davies et al. used absorbance difference spectrophotometry and found that bilirubin fluoresced when in the presence of either human or bovine serum albumin [26]. Bilirubin has a similar binding affinity to albumin from different species, indicating that it is a conserved mechanism for blood transport [27]. Gordon et al. used the autofluorescent property of bilirubin to show that bilirubin binds to PPARα [15], indicating that the autofluorescent property can be used for detecting other proteins bound. Binding was demonstrated using a non-fluorescent PPARα ligand, which developed a form of a non-radioactive competitive binding assay that utilized bilirubin’s autofluorescent properties similar to radioactive and non-radioactive competitive binding assays [15]. The non-radioactive competitive binding assay was further used to identify the amino acids required for bilirubin to bind in the PPARα ligand-binding pocket and that bilirubin did not bind PPARγ [28]. However, it is unknown whether urobilin has autofluorescent properties similar to bilirubin or if it binds albumin for transport in circulation.

In this study, we investigated whether urobilin binds albumin and has autofluorescent properties similar to bilirubin. We utilized in silico docking analysis to fit bilirubin and urobilin in the binding pocket of albumin, and autofluorescent assays were used to determine if urobilin binds to albumin in vitro. We found that urobilin likely binds albumin and has a similar autofluorescent property as bilirubin when it binds to a protein. This is the first study to show this property of urobilin and its binding to albumin.

## 2. Materials and Methods

### 2.1. Reagents

Phosphate-buffered saline (PBS) (Alkali Scientific, Fort Lauderdale, FL, USA), bovine serum albumin (BSA) (Fischer Scientific, Hampton, NH, USA), unconjugated bilirubin (EMD Millipore Corp, Burlington, MA, USA), urobilin (Frontier Scientific, Newark, DE, USA), and dimethyl sulfoxide (DMSO) (MP Biomedicals, Solon, OH, USA) were used in this study.

### 2.2. Chemical Structures

**Figure 1:**
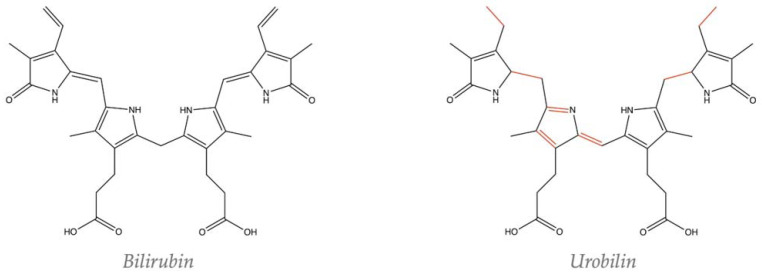
The bioconversion of bilirubin to urobilin alters the chemical structure. The red bonds in urobilin denote those that are different compared to bilirubin.

The chemical structures in Figure 1 were drawn using ChemDraw version 22.2.0.3348 (Revvity Signals Software, Waltham, MA, USA).

### 2.3. Molecular Docking Analysis

The initial structures of the ligands used in the present study were sketched and prepared at a pH of 7.4 using the LigPrep module of Schrödinger 2024-1 [22,23]. This ligand preparation procedure includes generating all the possible tautomeric and ionization states and a ligand’s stereoisomers (if any) [24]. All the generated ligand conformers were minimized using the OPLS4 force field [25]. Energy minimization involves the generation of the low-energy conformer of a ligand by the addition of hydrogen, assigning bond lengths and bond angles. The generated conformers were used for molecular docking studies.

The crystal structure of human serum albumin (PDB ID: 1BM0) was prepared for molecular docking using the Protein Preparation Wizard of Schrödinger 2024-1 [26,27]. This process ensures the structural correctness of a protein with high confidence accuracy. Protein preparation includes the assignment of bond orders, removal of water molecules, the addition of hydrogens, ionization of amino acids at a pH of 7.4, optimization of hydrogen bonds, and energy minimization of the protein. The generated protein state was used for the docking studies.

The prepared structures of ligands and protein underwent docking studies using the Glide module of Schrödinger 2024-1 [28]. Glide employs a grid-based docking protocol, where a grid is generated encircling the protein’s binding pocket. Scaling factor 1.0 was fixed for the van der Waals radii of non-polar atoms and 0.25 for the partial charge cut-off. Extra precision (XP) docking was performed, ensuring the highest accuracy level in studying the protein–ligand interactions within the available options provided by the software. The XP descriptors generated in this study were used to analyze the docking results.

### 2.4. General Autofluorescence Assay Setup

The autofluorescence assay was performed as previously described in [15,28]. Briefly, in a black flat-bottom 96-well plate, bovine serum albumin (BSA) (final concentration 50 µM), bilirubin (final concentration 50 µM), urobilin (final concentration 50 µM), DMSO, and/or PBS was added to the wells. Once compounds were added to the plate, the plate was protected with foil to avoid light degradation. The excitation and emission spectra of the samples were measured using the top-read Varioskan LUX Plate Reader (Thermo Scientific, Waltham, MA, USA). The samples were read in 5 nm steps with excitation and emission filters. The excitation spectra were recorded from 300 to 495 nm at emission 520 nm. Once the maximal excitation for urobilin bound to the albumin was recorded, the wavelength of the peak was set at the excitation value (490 nm) and used for the excitation wavelength for the subsequent emission spectrum from 510 to 700 nm.

### 2.5. Concentration–Response Autofluorescence Assay Setup

The concentration–response autofluorescence assay was performed as described above in Section 2.4 and as described previously [15,28]. An increasing concentration of urobilin was used from 0.78 to 300 μM. The plate was read over emission spectra from 510 to 700 nm at excitation 490 nm.

### 2.6. Competitive Binding Autofluorescence Assay Setup

The competitive binding assay was performed as described above in 2.4 and as described previously [15,28]. The amount of bilirubin and urobilin were adjusted to determine if they bind competitively. For both bilirubin and urobilin, 100% = 50 μM, 75% = 37.5 μM, 50% = 25 μM, and 25% = 12.5 μM. The plate was read over the emission spectra from 510 to 700 nm at excitation 490 nm.

### 2.7. Statistics

The data were graphed and analyzed via Prism 10 GraphPad Prism version 10.3.0 (GraphPad Software, La Jolla, CA, USA). Results are expressed as mean ± SEM. One-way ANOVA, with the least significant difference post hoc test was used to compare the area under the curve for each condition. *p* < 0.05 was considered statistically significant.

## 3. Results

### 3.1. Structural Difference Between Bilirubin and Urobilin

After being produced, bilirubin travels through systemic circulation primarily bound to albumin. Bilirubin is conjugated in the liver by UGT1A1 and transported into the intestines through bile for excretion. Once in the intestines, bacteria species that harbor the bilirubin reductase enzyme remove the conjugation and produce urobilinogen rapidly oxidized to urobilin (Figure 1). The alternation in the chemical structure changes its chemical properties, whereas urobilin is more hydrophilic than bilirubin. Urobilin is then reabsorbed into circulation through the hepatic portal vein. However, it is unknown if urobilin binds albumin to be transported in circulation similar to that of bilirubin.

### 3.2. Molecular Docking of Bilirubin and Urobilin to Albumin

**Figure 2:**
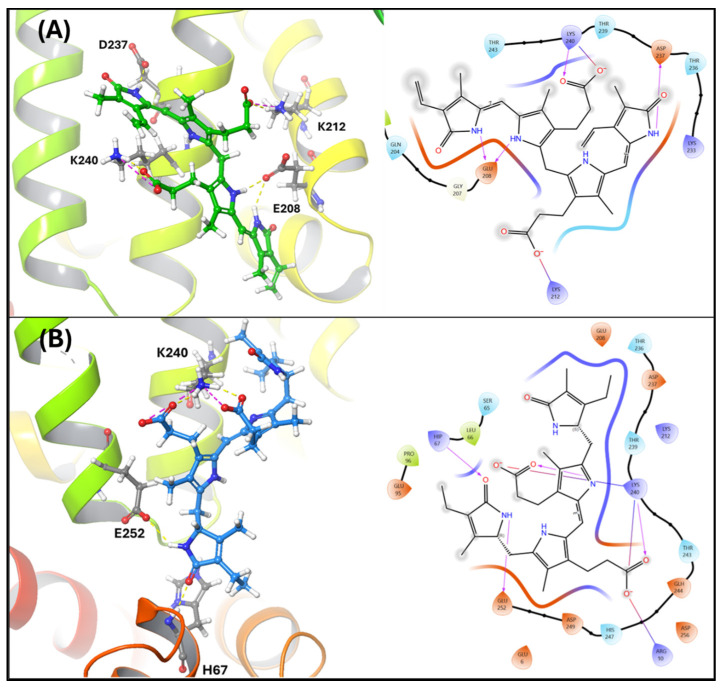
Bilirubin and urobilin interaction with albumin. (**A**) Bilirubin (green) in the binding pocket of human serum albumin. (**B**) Urobilin (blue) in the binding pocket of human serum albumin. The left half of the panels shows the 3D orientation, and the right half displays the 2D interaction plot. In the left panels, the yellow lines represent hydrogen bonds, and the purple lines indicate salt bridges.

In silico modeling was used to dock bilirubin and urobilin into the binding pocket of human serum albumin, which contains two primary binding sites, named site I and site II [29]. Our in silico docking analysis predicted bilirubin binds to albumin through four hydrogen bonds at D237, K212, E208, and K240 (Figure 2A). In addition to the hydrogen bonds, bilirubin forms salt bridges with K212 and K240 of albumin. We then used our analysis to dock urobilin with human albumin. Urobilin was predicted to bind albumin through three hydrogen bonds at H67, K240, and E252 and a salt linkage with K240 (Figure 2B). Interestingly, K240 interacts with both bilirubin and urobilin.

### 3.3. Autoflourescent Property of Urobilin When Bound to Albumin

**Figure 3:**
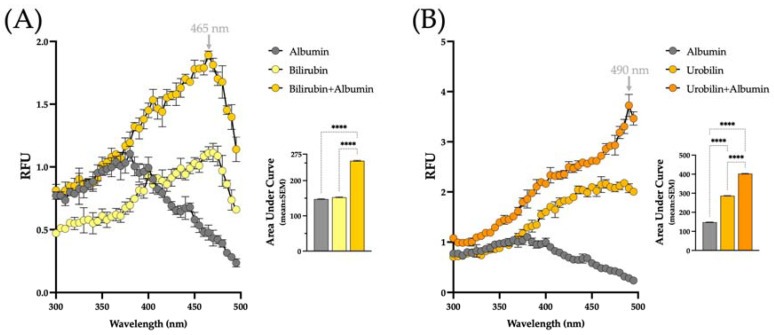
Urobilin and bilirubin present autofluorescence when bound to albumin. (**A**) Bilirubin (50 μM) binding to albumin (50 μM) excitation spectra with emission at 520 nm. (**B**) Urobilin (50 μM) binding to albumin (50 μM) excitation spectra with emission at 520 nm. The area under the curve was determined and graphed. **** *p* < 0.0001, one-way ANOVA. Each point represents three independent replicates and is displayed as mean ± SEM.

The autofluorescent properties of bilirubin and urobilin were utilized to investigate the binding to albumin in vitro. The autofluorescent property of bilirubin has been well described, whereas bilirubin alone does not fluoresce. Once albumin or another bilirubin-binding protein, such as PPARα, is added, bilirubin autofluorescence increases the fluorescence intensity (RFU) (Figure 3A). The binding was measured over excitation spectra at an emission of 520 nm, which is in line with the previous literature [15,28]. The area under the curve (AUC) was calculated and graphed. Bilirubin + albumin had a significantly higher area compared to albumin and bilirubin alone. The autofluorescence assay was repeated using urobilin instead of bilirubin to determine if it has a similar property and can bind to albumin. Albumin alone had no fluorescence. However, urobilin alone had a marginal background fluorescence. This fluorescence was intensified when urobilin was incubated with albumin (Figure 3B). The AUC was significantly higher in the urobilin + albumin than in the two alone. This indicates that urobilin has a similar autofluorescence property to bilirubin, which fluoresces when bound to protein and urobilin binds albumin. Both bilirubin and urobilin binding to albumin were measured over an excitation spectrum reading at emission wavelength 520 nm. The fluorescent intensity of bilirubin and urobilin peaked at different excitation wavelengths, 465 nm for bilirubin and 490 nm for urobilin, which is consistent with the previous fluorescent studies for bilirubin [15,28] and urobilin [30,31].

### 3.4. Concentration Response of Urobilin Binding to Albumin

**Figure 4:**
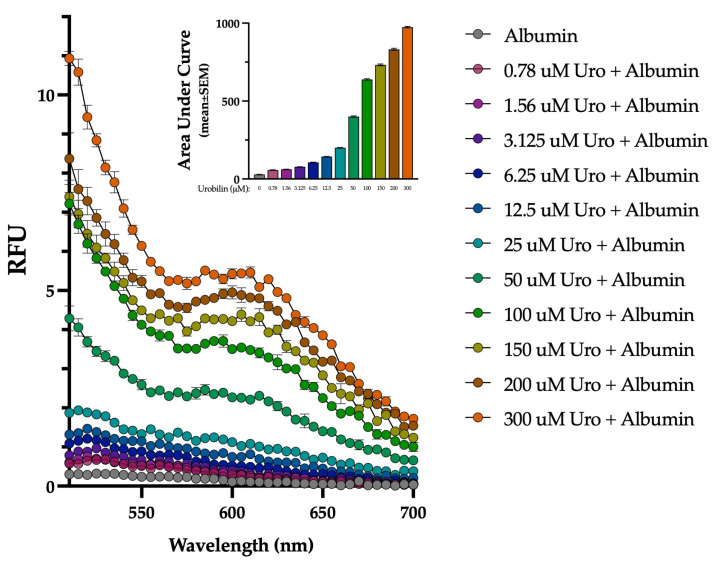
The fluorescent intensity of urobilin binding to albumin is concentration dependent. Binding assay with increasing concentrations of urobilin and albumin (50 μM) at excitation 490 nm. Each point represents three independent replicates and is displayed as mean ± SEM.

We designed a urobilin–albumin concentration–response experiment to explore the binding relationship between urobilin and albumin. We measured the urobilin concentration response to the binding using an emission spectrum at an excitation of 490 nm, which was determined based on the peak in Figure 3. As the urobilin concentration increased from 0.78 to 300 μM, we observed a higher fluorescent peak for the urobilin + albumin interaction (Figure 4). This indicates the urobilin-albumin binding relationship is concentration-dependent.

### 3.5. Bilirubin Competes with Urobilin for Binding to Albumin

**Figure 5:**
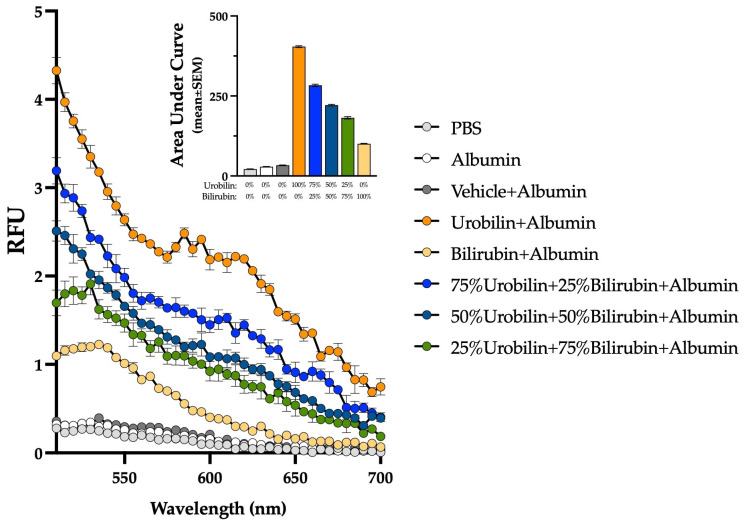
The combination of bilirubin and urobilin and autofluorescence. A competitive binding assay using different percentages of bilirubin and urobilin with albumin (50 μM). For bilirubin and urobilin, 100% = 50 μM, 75% = 37.5 μM, 50% = 25 μM, and 25% = 12.5 μM. Each point represents three independent replicates and is displayed as mean ± SEM.

Finally, a competitive binding assay was performed using different percentages of bilirubin and urobilin with albumin to determine if it altered the amount of fluorescence measured. We found that fluorescence intensity decreased when bilirubin was incubated with urobilin and albumin compared to only urobilin and albumin (Figure 5). Future investigations are needed to determine if they bind together or have secondary binding sites.

### 3.6. Graphical Conclusion

**Figure 6:**
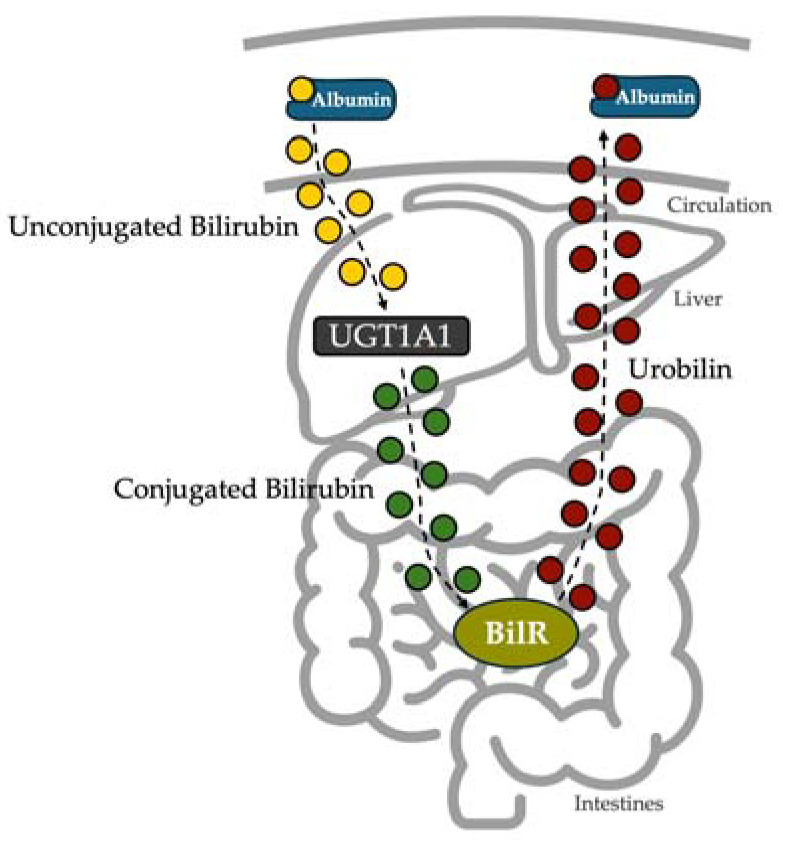
Urobilin is produced from the bioconversion of bilirubin and binds albumin for transport in circulation. Bilirubin is conjugated in the liver, transported to the intestines, and catabolized into urobilin. Urobilin can be reabsorbed into systemic circulation through the hepatic portal vein, which binds albumin for transport through the systemic circulation.

The data above indicate that urobilin binds to albumin. Urobilin is formed in the intestines, where microbiota expresses the BilR enzyme. This enzyme catabolizes bilirubin to form urobilinogen, which is then oxidized into urobilin. Urobilin enters circulation through the hepatic port vein, where it binds albumin (Figure 6).

## 4. Discussion

The significance of our study is that it is the first to identify that urobilin binds to albumin and that bilirubin and urobilin are likely to bind to different albumin amino acids. There is no known physiological function or receptor for urobilin. Almost a century ago, urinary urobilin/urobilinogen was positively associated with heart failure [32,33,34,35,36,37,38]. More recent literature surrounding urobilin is primarily derived from non-targeting mass spectrometry studies [19,20,39]. In humans, urobilin has been positively correlated with increased visceral fat area [19], all-cause mortality in diabetic patients [20], and heart failure [39]. A study by Smith et al. found that urobilin was positively associated with cardiovascular disease mortality, overall mortality, type 2 diabetes, and stroke, and its levels decreased with a health-conscious dietary pattern [40]. A study using untargeted metabolomics found that converting bilirubin to urobilin is a biomarker for chronic chagas cardiomyopathy in humans [41]. In a study on obese and lean humans, we identified that plasma urobilin was positively associated with adiposity and insulin resistance [13]. In animal models, urobilin was positively associated with acute myocardial ischemia [21] and diet-induced obesity [42]. Future studies are needed to determine urobilin’s mechanisms and diseases it’s associated with.

The inverse roles of urobilin and bilirubin in the cardiovascular–kidney–metabolic (CKM) syndrome have been discussed [2], and the two molecules may have different functions. Bilirubin’s function is likely the opposite of urobilin, where it protects against cardiometabolic dysfunction [9,43]. Bilirubin functions as an antioxidant [10] and a hormone through PPARα to activate fat-burning pathways [11,44]. In animal models of diet-induced obesity, bilirubin nanoparticles lower adiposity [15], MASLD [14], and hepatic ceramide production [13,14,15]. Bilirubin nanoparticles also protect against cardiac ischemia/reperfusion injury [45] and atherosclerosis [46].

Enzymes that produce bilirubin, such as biliverdin reductase A (BVRA), [47] are essential to liver function as a loss in hepatocytes causes lipid accumulation and oxidative stress [48,49,50], which also occurs in kidney cells [51]. However, it has been demonstrated that blocking UGT1A1, specifically in the liver, using an RNAi targeted to the liver, caused an increase in bilirubin and a reduction in plasma urobilin [52]. The UGT1A1-RNAi lowered plasma urobilin levels, adiposity, glucose intolerance, hepatic lipid accumulation, and pro-inflammatory kinase signaling pathways, which suggests targeting UGT1A1 to control urobilin levels as a potential therapeutic [52,53]. Whether bilirubin and urobilin have similar roles in hepatic disease is unknown. More investigations on their functions in liver fibrosis [54] and insulin resistance [55] are needed to better understand their roles, as the pathways they control might be diverse.

Bilirubin requires albumin as a carrier protein to travel through systemic circulation, but whether urobilin also binds to albumin was unknown until this study. Our in silico docking analysis showed that bilirubin and urobilin bind to albumin. We found that bilirubin interacts with D237, K212, E208, and K240 in site I of albumin. Previous in vitro studies determined that K240 is responsible for bilirubin’s high affinity towards human albumin [56]. This site is slightly shifted in bovine albumin, where residues 186–238 are important for bilirubin binding [57]. Bilirubin’s carboxyl tail interacts with K240 and, interestingly, we found that urobilin also interacts with K240 of human albumin. The carboxyl tails are conserved between bilirubin and urobilin, which may explain why they interact with K240. There were no other residues in common between urobilin and bilirubin. In addition to K240, urobilin interacted with H67 and E252 of human albumin through hydrogen bonds, indicating strong binding.

When bilirubin binds to a protein and is excited by light, it autofluoresces, which can be measured [23,24]. The bilirubin–PPARα interaction has been investigated through the use of bilirubin’s autofluorescent property. Adeosun et al. showed that biliverdin to bilirubin could be measured by using the UnaG fluorescent protein from the unagi eel [58] to quantify unconjugated bilirubin levels. Gordon et al. used bilirubin’s autofluorescence from light excitement to show that it binds to PPARα and elicits a similar autofluorescence as when it binds to albumin [15]. Using site-directed mutagenesis, mutations were made at the predicted bilirubin binding sites of PPARα, and we found that mutating amino acids T283 and A333 of PPARα significantly reduced the fluorescence intensity [28]. We applied the same autofluorescence assay to investigate if urobilin binds to albumin. We found that although urobilin had background fluorescence by itself, the fluorescent intensity significantly increased when combined with albumin. This indicates that urobilin binds to albumin and autofluoresces, similar to bilirubin. When comparing the excitation spectrum, bilirubin and urobilin peaked at different wavelengths, 465 nm and 490 nm, respectively. To date, this is the only urobilin-binding protein that has been identified.

The physiological importance of urobilin binding to albumin is still unknown and requires more studies. Urobilin may use albumin strictly as a carrier protein, but this interaction may also change the pharmacology of urobilin binding to its receptor if one is uncovered or it might interfere with bilirubin binding albumin. The binding of molecules to albumin has been shown to increase their half-life [59]. The binding of urobilin to albumin may extend its half-life by preventing its urinary excretion, but this still requires more testing. Also, aspects of urobilin in physiology, such as whether its plasma levels change with exercise like bilirubin [60,61,62] needs further investigation. It is possible that urobilin levels might decrease with exercise based on its association with increased adiposity and insulin resistance in humans [13,63]. Future studies should investigate the relationship between plasma and urinary urobilin with albumin levels, urobilin with the metabolic diseases described above, and other conditions in which bilirubin has been found to be a positive regulator [64], as urobilin may have an opposing role.

## 5. Conclusions

In conclusion, this work has identified albumin as the first protein shown to bind urobilin. We also show that urobilin has an autofluorescent property like bilirubin, and both autofluoresce when bound to protein but at different peak wavelengths. Future work is needed to elucidate the physiological function of urobilin and if it has a definitive role in disease(s), especially CKM syndrome. The findings of our investigation are an initial step in understanding urobilin and its importance in physiology and disease pathology.

## Data Availability

The original contributions presented in the study are included in the article, further inquiries can be directed to the corresponding authors.
